# Supplementary material for: Human thymopoiesis produces polyspecific CD8+ α/β T cells responding to multiple viral antigens
Source: eLife. 2023 Mar 30;12:e81274. doi: 10.7554/eLife.81274 (PMC10063231; doi:10.7554/eLife.81274)
Supplement: Figure 2—source data 1. [file elife-81274-fig2-data1.docx]

| **Patient** | **Publicness** | **DP** | **CD8** | **p-value** | **Odds ratio** |
| --- | --- | --- | --- | --- | --- |
| **P11** | Public | 590 | 1630 | p<0.0001 | 0.3403 |
|  | Private | 17410 | 16370 |  |  |
| **P18** | Public | 695 | 1567 | p<0.0001 | 0.4212 |
|  | Private | 17305 | 16433 |  |  |
| **P19** | Public | 485 | 1489 | p<0.0001 | 0.3071 |
|  | Private | 17515 | 16511 |  |  |
| **P23** | Public | 649 | 1489 | p<0.0001 | 0.4148 |
|  | Private | 17531 | 16511 |  |  |
| **P24** | Public | 578 | 1416 | p<0.0001 | 0.3886 |
|  | Private | 17422 | 16584 |  |  |
| **P25** | Public | 640 | 1536 | p<0.0001 | 0.3952 |
|  | Private | 17360 | 16464 |  |  |
| **P26** | Public | 640 | 1536 | p<0.0001 | 0.3771 |
|  | Private | 17360 | 16464 |  |  |
| **P27** | Public | 640 | 1583 | p<0.0001 | 0.3823 |
|  | Private | 17360 | 16417 |  |  |
| **P29** | Public | 655 | 1583 | p<0.0001 | 0.3916 |
|  | Private | 17345 | 16417 |  |  |
| **P36** | Public | 653 | 1468 | p<0.0001 | 0.4239 |
|  | Private | 17347 | 16532 |  |  |

**Figure 2 - source data 1. Enrichment of public βCDR3s in CD8^+^ thymocytes vs DPCD3^+^.**

Contingency table for the Chi-square analysis performed with Yates' correction to test the null hypothesis of independence between the sharing of βCDR3s (Public or Private) vs the cell phenotype (DPCD3^+^ and CD8^+^). We performed this test in the 10 donors for which we have 18,000 βCDR3s in both DPCD3^+^ and CD8^+^ thymocytes. A βCDR3 is defined as public if it is found at least once in the 18,000 βCDR3s of the same cell phenotype from other donors. The results (p-value < 0.0001) rejected the null hypothesis, thereby indicating the interdependency of the two variables.
