# Supplementary material for: Human thymopoiesis produces polyspecific CD8+ α/β T cells responding to multiple viral antigens
Source: eLife. 2023 Mar 30;12:e81274. doi: 10.7554/eLife.81274 (PMC10063231; doi:10.7554/eLife.81274)
Supplement: Figure 3—source data 1. [file elife-81274-fig3-data1.docx]

| **Patient** | **Publicness** | **DP** | **CD8** | **p-value** | **Odds ratio** |
| --- | --- | --- | --- | --- | --- |
| **P11** | Virus Tet+ | 106 | 8 | p<0.0001 | 40.75 |
|  | Unknown | 4389 | 13497 |  |  |
| **P18** | Virus Tet+ | 126 | 10 | p<0.0001 | 39.93 |
|  | Unknown | 4285 | 13579 |  |  |
| **P19** | Virus Tet+ | 102 | 8 | p<0.0001 | 46.77 |
|  | Unknown | 3832 | 14058 |  |  |
| **P23** | Virus Tet+ | 93 | 7 | p<0.0001 | 47.82 |
|  | Unknown | 3892 | 14008 |  |  |
| **P24** | Virus Tet+ | 96 | 9 | p<0.0001 | 40.38 |
|  | Unknown | 3739 | 14156 |  |  |
| **P25** | Virus Tet+ | 112 | 7 | p<0.0001 | 63.60 |
|  | Unknown | 3594 | 14287 |  |  |
| **P26** | Virus Tet+ | 83 | 2 | p<0.0001 | 146.1 |
|  | Unknown | 3963 | 13952 |  |  |
| **P27** | Virus Tet+ | 99 | 8 | p<0.0001 | 34.60 |
|  | Unknown | 4714 | 13179 |  |  |
| **P29** | Virus Tet+ | 55 | 9 | p<0.0001 | 31.65 |
|  | Unknown | 2903 | 15033 |  |  |
| **P31** | Virus Tet+ | 104 | 1 | p<0.0001 | 360.8 |
|  | Unknown | 4004 | 13891 |  |  |
| **P32** | Virus Tet+ | 44 | 9 | p<0.0001 | 32.93 |
|  | Unknown | 2320 | 15627 |  |  |
| **P36** | Virus Tet+ | 86 | 6 | p<0.0001 | 54.12 |
|  | Unknown | 3750 | 14158 |  |  |

**Figure 3 – source data 1. Enrichment of virus-specific ßCDR3s from databases14,15 in clustered CD8+ thymocytes.**

Contingency table for the Chi-square analysis performed with Yates' correction to test the null hypothesis of independence between the specificity of βCDR3s (Virus Tet+ and Unknown specificities) vs the connection of βCDR3 (“clustered” and “dispersed”) in all the CD8+ thymocytes from 12 donors. The results (p-value < 0.0001) rejected the null hypothesis, thereby indicating the interdependency of the two variables.
