# Supplementary material for: Human thymopoiesis produces polyspecific CD8+ α/β T cells responding to multiple viral antigens
Source: eLife. 2023 Mar 30;12:e81274. doi: 10.7554/eLife.81274 (PMC10063231; doi:10.7554/eLife.81274)
Supplement: Figure 4—source data 1. [file elife-81274-fig4-data1.docx]

| **Peptide** | **Virus** |
| --- | --- |
| FLRGRAYGL | EBV |
| FLYALALLL | EBV |
| GLCTLVAML | EBV |
| LLDFVRFMGV | EBV |
| RAKFKQLL | EBV |
| RTLNAWVKV | HIV |
| SLFNTVATL | HIV |
| SLFNTVATLY | HIV |
| MLDLQPETT | HPV |
| LLFGYPVYV | HTLV |
| GILGFVFTL | Influenza |
| ELRRKMMYM | CMV |
| VTEHDTLLY | CMV |
| SLYNTVATLY | HIV |

**Figure 4 – source data 1. List of peptides represented on the chord plot from Figure 4C right.**

The table is organized according to the clockwise order of the chord plot segments.
