# Supplementary material for: Human thymopoiesis produces polyspecific CD8+ α/β T cells responding to multiple viral antigens
Source: eLife. 2023 Mar 30;12:e81274. doi: 10.7554/eLife.81274 (PMC10063231; doi:10.7554/eLife.81274)
Supplement: Figure 5—source data 1. [file elife-81274-fig5-data1.docx]

| **TCR** | **chain** | **V_gene** | **D_gene** | **J_gene** | **C_gene** | **CDR3** |
| --- | --- | --- | --- | --- | --- | --- |
| **#35-13** | TRA | TRAV3 | None | TRAJ45 | TRAC | CAVAKDLQNSGGGADGLTF |
|  | TRB | TRBV19 | None | TRBJ2-7 | TRBC2 | CASSARSSTEQYF |
| **#36-150** | TRA | TRAV27 | None | TRAJ42 | TRAC | CAGAGSQGNLIF |
|  | TRB | TRBV19 | None | TRBJ2-7 | TRBC2 | CASSIRSSYEQYF |
| **D222D-ZnT8186-194** | TRA | TRAV17 | None | TRAJ36 | TRAC | CAVTGANNLFF |
|  | TRB | TRBV19 | TRBD1 | TRBJ2-2 | TRBC2 | CASSIEGPTGELF |
| **GAD 114-122** | TRA | TRAV21 | None | TRAJ26 | TRAC | CAVGDNYGQNFVF |
|  | TRB | TRBV1 | TRBD1 | TRBJ1 | TRBC2 | CASSISPFNGVGSPLHF |

**Figure 5 – source data 1. List of the sequences of the individual TCRs expressed and analyzed in Figure 5**
